# Time from symptom onset may influence C-reactive protein utility in the diagnosis of bacterial infections in the NICU

**DOI:** 10.1186/s12887-022-03783-4

**Published:** 2022-12-14

**Authors:** Shelley Borowski, Irina Shchors, Maskit Bar-Meir

**Affiliations:** 1 Pediatrics Department, Shaare-Zedek Medical Center, Jerusalem, Israel; 2 Neonatal Intensive Care Unit, Shaare-Zedek Medical Center, Jerusalem, Israel; 3 Pediatric Infectious Diseases, Shaare-Zedek Medical Center, P.0.B 3235, 91301 Jerusalem, Israel; 4 The Faculty of Medicine, Hebrew University, Jerusalem, Israel

**Keywords:** C-reactive protein, Neonatal intensive care unit, Bacterial infection

## Abstract

**Background:**

Taking into account the timing of C-reactive protein (CRP) testing may improve the performance of the test in diagnosing bacterial infections in the neonatal intensive care unit (NICU). We aimed to examine the yield of CRP, relative to time from symptoms onset.

**Methods:**

Enrolled were all NICU patients, for whom CRP was obtained as part of a sepsis workup. The time of symptoms onset and of blood draw was recorded. Patients were classified into bacterial and non-bacterial groups according to the National Healthcare Safety Network (NHSN) guidelines. The performance of CRP, CRP velocity, and CRP obtained before or after 6 hours from symptoms onset, was evaluated by receiver-operating characteristic (ROC) curve. Test characteristics were calculated using formulas based on Bayes’ theorem.

**Results:**

Of 129 infants enrolled in the study, 21(16%) had a bacterial infection. A single CRP test and CRP velocity performed similarly in diagnosing bacterial infection, with area under ROC curve of 0.75 (95%CI: 0.61–0.89) and 0.77 (95% CI:0.66–0.88), respectively. The optimal cut-off value for a CRP test obtained <= 6 hours from symptoms onset was 1 mg/dL, whereas the optimal cut-off > 6 hours was 1.5 mg/dL. Using the optimal cut-off values increased the pre-test probability of 16%, to a post-test probability of 35–38%. For infants whose birth weight was < 1000 g, CRP performed poorly.

**Conclusions:**

The optimal CRP cut-off used in the diagnosis of bacterial infections in NICU patients varies by the time from symptom onset. A “negative” CRP may support a clinical decision to stop empiric antimicrobial therapy, for infants whose blood cultures remain sterile.

**Supplementary Information:**

The online version contains supplementary material available at 10.1186/s12887-022-03783-4.

## Background

Neonatal sepsis is associated with substantial morbidity and mortality [1, 2].

The signs and symptoms of neonatal sepsis may be subtle and non-specific, making a timely diagnosis of sepsis difficult. As a result, broad-spectrum antibiotics are frequently prescribed in the neonatal intensive care unit (NICU), while awaiting culture results. This widespread use of antimicrobials is associated with the emergence of multidrug-resistant organisms, therefore tools to support antibiotic stewardship are urgently needed [3]. Several diagnostic tests, such as C–reactive protein (CRP), procalcitonin, and interleukin- 6, have been evaluated as aids for the diagnosis of neonatal sepsis. Of these, CRP is the most extensively studied acute phase reactant. It is widely available, simple, fast,and cost-effective [4]. The hepatic synthesis rate of CRP increases within hours after the onset of infection and can increase up to 1000-fold from baseline levels. CRP levels remain high while tissue damage or inflammation persists, and then falls rapidly [4]. Elevated CRP levels in the neonate always represent endogenous synthesis, since CRP passes the placenta in extremely low amounts [5]. The physiologic kinetics of CRP after birth has to be taken into account when interpreting CRP levels. Shortly after birth, there is a CRP rise related to the stress of delivery. Chiesa et al. demonstrated that the 95th percentiles of CRP values in healthy neonates were different at birth, 24 and 48 hours of life [6].

Therefore, a fixed CRP cut-off value, especially for diagnosing early-onset sepsis, may ignore these physiologic changes.

Moreover, CRP response is influenced by gestational age and birth weight. It has been demonstrated that CRP has a lower sensitivity for sepsis diagnosis in preterm compared to term newborn [7], and that CRP rise induced by infection in preterm infants is significantly lower and with shorter duration [6]. The timing of CRP testing relative to the onset of infection may also influence CRP peak levels. CRP sensitivity is lowest during the early stages of infection [7] and increases dramatically within 24–48 hours after the onset of symptoms [8]. Obtaining serial CRP, rather than a single test, over the first 24–48 hours from onset of symptoms, increases the negative predictive value (e.g identifying infants without sepsis) to 99% [4]. Since serial testing in neonates is difficult, and the information obtained from a single test is limited [9], the concept of CRP velocity- the rate of CRP rise - has been suggested as a tool that would obviate the need to take multiple blood tests, yet take into account the time from symptoms onset. We have previously shown that the optimal cut-off CRP value for the diagnosis of bacterial infection, among children presenting to the emergency department (ED), changed with time from fever onset. Interpreting the CRP results in the context of time from fever onset, significantly improved the yield of the test [9].

Here, we performed a prospective observational study to examine the yield of the CRP increase rate (e.g “CRP velocity”) for diagnosing bacterial infections in the NICU and to define the optimal CRP cut-off values, relative to the time from symptoms onset.

## Methods

The NICU of Shaare-Zedek Medical Center (SZMC), an academic tertiary care center, is a 70-bed level III NICU, that serves the Jerusalem area. SZMC has > 20,000 in-hospital deliveries annually, and the NICU also serves as a referral center for complicated medical or surgical cases from east-Jerusalem NICUs. The NICU has on average 1300 admissions/year, totaling an average of 19,000 annual patient-days.

### Sample size estimation

In a previous study, performed in our pediatric ED (*N* = 373), we found the mean CRP velocity in the bacterial group to be 0.77 ± 1.6 mg/dL/h compared with 0.18 ± 0.31 mg/dL/h in the viral group. Every month, approximately 40–50 sepsis evaluations are performed in our NICU, of which approximately 10% are diagnosed as bacterial infections. Therefore, a sample size of 120 patients would provide a power of 80% to detect at least a 50% difference in CRP velocity in infants with and without bacterial infection, with a level of significance of 0.05.

This was a prospective observational study. All NICU patients, for whom CRP was obtained as part of their sepsis workup, were enrolled. CRP was obtained at the discretion of the treating physicians. No tests were performed for the purpose of the study. The study team was not involved in the evaluation or treatment of any infant enrolled in this study. Subsequent CRP tests, obtained at the discretion of the treating physician, were recorded as well.

The exact time of symptoms onset was determined according to the nurses’ notes. If several signs/ symptoms occurred, the earliest one was used to determine the onset. Time was recorded in hours. The time of blood draw for CRP testing was collected from nurses’ notes and based on the time recorded on the test order forms. The time of symptoms onset was determined in a blinded fashion before CRP results were known.

One of the authors (M.B-M) has divided the patients into 2 main groups:1.Bacterial infections or 2. No apparent bacterial infections. Both early(< 72 hours) and late-onset bacterial infections were enrolled. Bacterial infections were defined according to the Centers for Disease Control and Prevention (CDC) National Healthcare Safety Network (NHSN) guidelines. When blood culture was positive for commensal organisms (bacillus, coagulase-negative staphylococci), the same commensal had to be isolated in > 2 specimens collected on separate occasions for the episode to be considered as a bacterial infection [10]. The classification of bacterial and non-bacterial groups was performed before knowledge of the CRP results.

Demographic and clinical characteristics were collected from patient charts. CRP tests (provided in mg/dL) were performed at the SZMC biochemistry laboratory, using 100 μL of blood, according to the manufacturer’s instructions (Vitros 5.1, Ortho Diagnostics, Johnson & Johnson, USA).

### Statistical analysis

Analysis was performed with SPSS software V.25 (SPSS, Chicago, Illinois, USA). Continuous variables were compared with t-test or Mann–Whitney U-test as appropriate. Categorical variables were compared with χ2 test. CRP velocity was calculated by dividing CRP value (mg/dL) by the time from symptoms onset (hours). For patients who had subsequent CRP tests over time, CRP velocity decrease was calculated as CRP velocity at time 1/CRP velocity at time 2. The diagnostic performance of CRP at different time points from symptoms onset and for patient subgroups (preterm infants < 36 + 6; < 1000 g; those who were on antimicrobial therapy) was compared between the bacterial and non-bacterial groups using a receiver-operating characteristic (ROC) curve. We determined sensitivity, specificity, and likelihood ratios (LRs) for the detection of bacterial infection using cut-off points that would maximize sensitivity without significantly compromising specificity (e.g, the point closest to the upper left corner of the ROC curve). Test characteristics, as well as the resulting post-test probabilities for bacterial infection, were determined for different durations of symptoms. For a given ROC curve, we used formulas based on Bayes’ theorem: the LR was calculated using the formula (positive LR = sensitivity/1 – specificity; negative LR = 1 – sensitivity/specificity) and the resulting post-test probability for bacterial infection (pretest probability/[1 – pretest probability])X LR/(pretest probability/[1 – pretest probability])X LR + 1 [11]. All reported *p*-values are two-sided and considered significant at *p* < 0.05.

A separate subgroup analysis was performed for patients who only partially met the CDC definitions for localized bacterial infections, such as necrotizing enterocolitis or pneumonia (for example, patients with bilious gastric aspirate and abnormal abdominal X-ray, but without frank pneumatosis intestinalis; new lung infiltrate without hemodynamic instability or complete blood count abnormalities [10]. These patients were defined as having “possible” bacterial infection, but did not meet the strict CDC definitions.

### Ethics

The study was approved by the SZMC Helsinky committee. Since no interventions were performed for the purpose of the study, and all data were de-identified, the committee waived the need to obtain informed consent.

## Results

Overall, 131 term and preterm babies were evaluated for sepsis and had CRP performed as part of their evaluation, between November 2014 and April 2015. For two patients, the blood culture results were not found in the database, they were therefore excluded. The remaining 129 patients were divided into two groups: bacterial and non-bacterial. The bacterial group included 21 patients (16%). Only one had early onset sepsis (occurring < 72 hours of age). Twelve had gram-positive bacteremia: 6 methicillin-resistant *S.aureus* (MRSA) bacteremia (one complicated with osteomyelitis and one with empyema); 5 with persistent coagulase-negative s*taphylococci* (CONS) bacteremia, and 1 with *Bacillus cereus* line infection. Nine patients had gram-negative bacteremia (*E.coli*, *Klebsiella pneumoniae, Acinetobacter baumanii).*

The most frequent symptoms leading to sepsis workup were apnea and respiratory deterioration, followed by gastrointestinal (GI) symptoms and fever. The presenting symptoms did not differ between the groups.

Table 1 describes the characteristics of the bacterial and non-bacterial groups. Patients in both groups were similar in their demographic characteristics. The time interval between CRP and symptoms was similar as well.

**Table 1:** Clinical and demographic characteristics of patients undergoing sepsis workup in the neonatal intensive care unit, according to bacterial or non-bacterial causes

	Bacterial (*N* = 21)	Non bacterial (*N* = 108)	*P* value
*Demographic characteristics*			
Gestational age, weeks Median (IQR)	28 (27,38)	32 (27,36)	0.1
Gestational weight, grams, Median (IQR)	1800 (1030,3037)	1925 (1125,2641)	0.9
Preterm (<=36 + 6 week of gestation), N (%)	20 (95%)	87 (80.5%)	0.3
Age at CRP in days, Median (IQR)	10.5 (5,68)	9.5 (2,25)	0.01
*Clinical characteristics*			
Interval between symptoms onset and CRP, hours, median (IQR)	3.5 (1.5,18)	5 (2.7,12)	0.8
Central Venous catheter, *N* (%)	8 (38)	22 (20)	0.09
Surgery prior to sepsis Workup, N(%)	7(33)	11 (10)	0.01 0.8
Absolute neutrophil count, cells/mm^3^,Median (IQR)	8.2 (1.9,16.4)	5.9 (2.5,9.3)	0.01
CRP value, mg/dL Median (IQR)	8 (1.3,21)	0.35 (0.3,1.5)	0.0001

^*^CRP C-reactive protein

*IQR* Interquartile range

### Single CRP test characteristics

The area under the ROC curve (AUC) for a single CRP test to diagnose bacterial infection in NICU patients was 0.75 (95%CI: 0.61–0.89, Fig. S1A). An absolute value of 1 mg/dL had a sensitivity of 76% and specificity of 70% to diagnose bacterial infection. When we examined separately extremely low birth weight infants (<=1000 g, *N* = 24; 6 with bacterial infections), the test performed worse: AUC 0.6, 95%CI: 0.2–0.9. In infants < 1000 g an absolute value of 1 mg/dL had a sensitivity of 50% and specificity of 76% to diagnose bacterial infection.

CRP performance was similar between infants who received antimicrobial therapy at the time of sepsis workup (*N* = 26; 6 with bacterial infections) and those who did not.

### CRP dynamics over time from fever onset

**Fig. 1:**
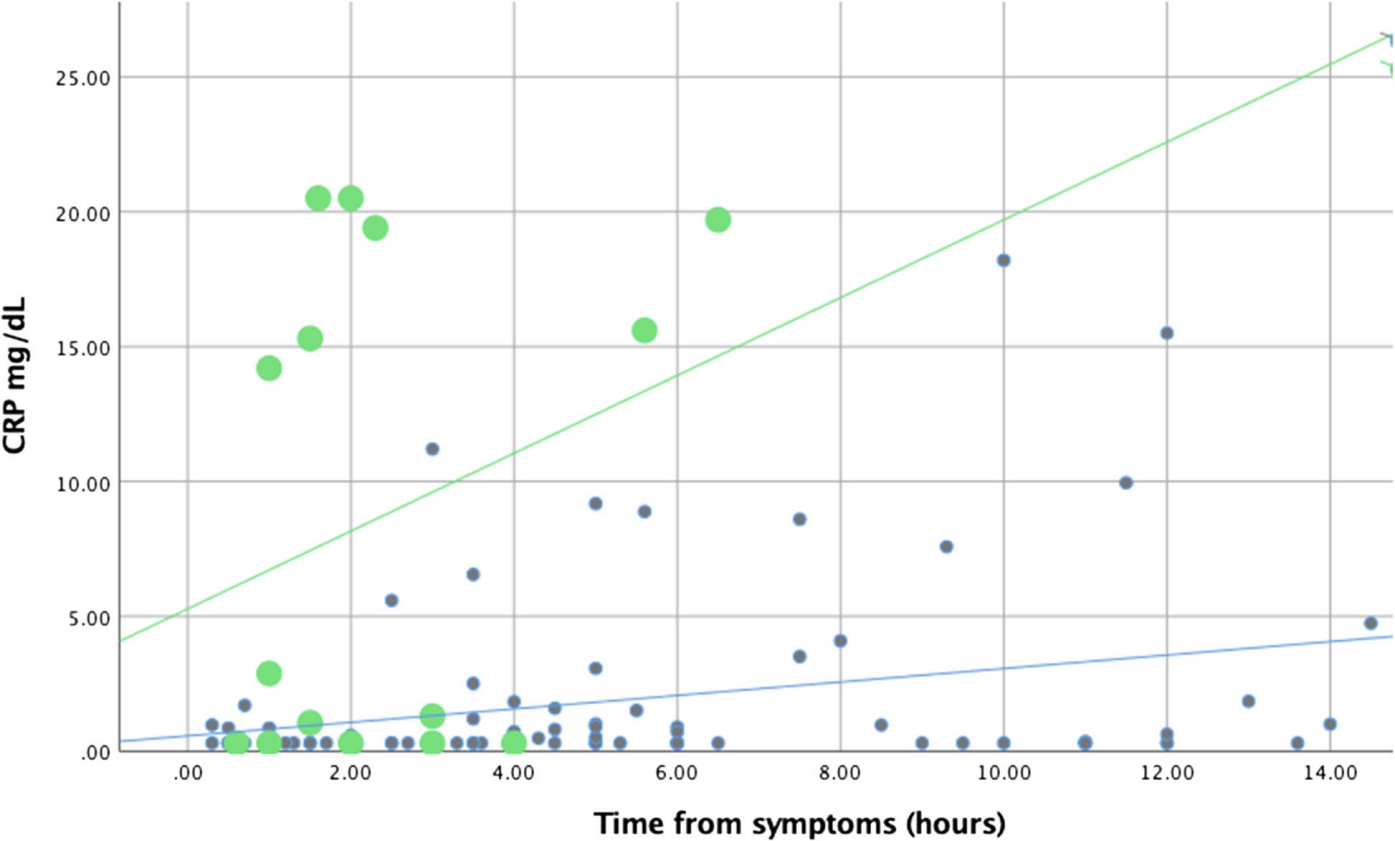
CRP values (mg/dL) according to time from symptoms onset. Large green dots- bacterial infections, small dark dots- non-bacterial. *P* value =0.0001 for bacterial vs. non-bacterial

CRP velocity was 3.3 ± 1 mg/dL/hr. in bacterial infections vs. 0.4 ± 0.6 mg/dL/hr. in non-bacterial infections (*p* = 0.0001, Fig. 1). The area under the ROC curve for CRP velocity to diagnose bacterial infection in NICU patients was 0.77 (95% CI:0.66–0.88, Fig. S1B). For 60 patients (15 bacterial, 45 non-bacterial), subsequent CRP tests were available. CRP velocity decrease over time was similar in bacterial vs. non-bacterial infections: 35-fold (±62) for bacterial infections vs. 34.5-fold (±88) for non-bacterial infections (*p* = 0.9, Fig. S2).

### CRP interpretation according to time from symptoms onset

The performance of the absolute CRP value to diagnose bacterial infection varied according to time from symptoms. Since most of the increase occurs within hours from symptom onset, we have examined the performance of CRP obtained <= 6 hours vs. > 6 hours from symptom onset. The optimal cut-off value for a CRP test obtained <= 6 hours from symptoms onset to diagnose bacterial infections, was 1 mg/dL, whereas the optimal cut-off > 6 hours from symptoms onset was 1.5 mg/dL (Table 2). CRP > 1.5 mg/dL, obtained > 6 hours from symptoms onset, was 100% sensitive to diagnose bacterial infection, at a cost of relatively low specificity. Using the optimal cut-off values according to the timing of CRP testing, CRP increased the pre-test probability of 16%, to a post-test probability of 35–38%.

**Table 2:** CRP performance <=6 hours vs. > 6 hours from symptoms onset in NICU patients undergoing sepsis workup

	AUC % (95%CI)	Sensitivity (95%CI)	Specificity (95%CI)	+LR (95%CI)	Post-test probability,% (95%CI)
<= 6 hrs *N* = 74 ^*^ **1 mg/dL**	0.7 (0.52,0.89)	64 (39–84)	80 (68–88)	3.2 (1.7–6)	38 (24–54)
> 6 hrs *N* = 51 ^*^1.5 mg/dL	0.87 (0.75,1)	100 (64–100)	64 (49–76)	2.8 (1.9–4)	35 (25–45)

Pretest probability = 16%

*CI* confidence interval, *AUC *area under the ROC curve, *CRP *C-reactive protein, *+LR *positive likelihood ratio

*Cut-off value

### CRP performance in the “possible” bacterial infection group

Twelve patients had suspected NEC (*N* = 8) or pneumonia (*N* = 4) but did not meet the strict definition of the CDC for bacterial infection. Mean CRP in these patients was 2.47 ± 3.3 mg/dL, the area under the ROC curve was 0.66 (95%CI: 0.66–0.8) and CRP of 1 mg/dL had only 50% sensitivity and 74% specificity to diagnose “possible” bacterial infection.

## Discussion

This study shows that the performance of CRP is influenced by the time elapsed from symptom onset. CRP velocity did not significantly improve CRP performance to diagnose bacterial infections in NICU patients- area under the ROC curve of 0.75 for CRP vs. 0.77 for CRP velocity. The rate of CRP decrease was also similar in bacterial vs. non-bacterial infections. However, we found that considering the time from onset of symptoms improved CRP performance, and changed the optimal test cut-off value: CRP > 1.5 mg/dL obtained > 6 hours from symptoms onset improved the area under the ROC curve to 0.87 and had 100% sensitivity to diagnose bacterial infections. The cost is a relatively low specificity of 64%. Adding the time factor improved the pre-test probability from 16 to 35%.

Prematurity (e.g gestational week < 36 + 6) did not change these findings. However, for extremely low birth weight infants < 1000 g, CRP performed poorly: CRP of 1 mg/dL had a sensitivity of 50% to diagnose bacterial infections.

The burden of morbidity and mortality due to neonatal sepsis in NICU patients remains high. However, blood culture-negative, clinically diagnosed sepsis accounts for the majority of these cases. Moreover, the definition of clinical sepsis is variable and often relies on subjective or non-specific signs on physical exam [12]. In fact, antibiotic use for rule-out sepsis episodes and culture -negative sepsis is 10–15 times higher than for culture-proven sepsis [13].

For a clinical condition with potentially grave consequences, such as neonatal sepsis, laboratory tests may support the treatment decisions, but have to be interpreted with caution. For example, according to our data, CRP of > 1.5 mg/dL, obtained > 6 hours from symptoms onset, in an infant who weighs > 1000 g, has 100% sensitivity to diagnose sepsis. It has been argued that for a highly sensitive test, a negative test essentially rules out the disease [14]. Unfortunately, the positive/negative likelihood ratios of the test are also influenced by the specificity [12], which in our example was relatively low. This translates to a negative predictive value for sepsis in this case of 65%. In other words, one cannot safely rule-out sepsis based on a “negative” CRP. On the other hand, once empiric antibiotic therapy is initiated, and blood cultures remain negative, a “negative” CRP may support a decision to stop antibiotics, and therefore may shorten the duration of antimicrobial use. These findings are in line with a recent large prospective study that showed, for early-onset sepsis, that if CRP remains below 1.6 mg/dL within 36 hours after the start of antibiotics, antibiotic treatment can be stopped safely [15].

The strength of this study was its prospective nature and blinded classification of the patients. To avoid verification bias, we used the strict definitions of the CDC NHSN guidelines to classify bacterial infections. For example, infants with GI symptoms and abnormal abdominal X-ray, but no bacteremia, were not classified as bacterial infections, but in real-life would probably be managed as necrotizing enterocolitis and receive a full course of antimicrobial therapy. At a subgroup analysis,we found that mean CRP in these patients with “possible bacterial infection was higher than in patients with no bacterial infection, but the overall performance of CRP was relatively poor. Therefore, in our cohort, CRP was not useful as an aid for the diagnosis of NEC or pneumonia, in the absence of bloodstream infection.

Our strict definitions led to the major limitation of our study- a relatively small patient sample. This may affect the study’s accuracy, as shown in the relatively wide confidence intervals of our findings. Another limitation is the fact that the performance of every test depends on whether the test is performed in a low or high-risk population. The interpretation of our results may therefore change between Level II and Level III nurseries, as well as between early-onset sepsis, which has a relatively low prevalence [16], and late-onset sepsis. Finally, the timing of symptom onset was determined by the nurses’ notes. This may be subjective and prone to inaccuracies.

*In conclusion*, the optimal cut-off for CRP to diagnose bacterial infections in NICU patients varies by the time from symptom onset. A cut-off of 1 mg/dL for CRP obtained < 6 hours from symptoms or 1.5 mg/dL if obtained after 6 hours increased the post-test probability from 16% to 35–38%. A “negative” CRP may support a clinical decision to stop empiric antimicrobial therapy for infants whose blood cultures remain sterile. In infants whose birth weight was < 1000 g, CRP performance was poor.

## Supplementary Information

**Additional file 1:** **Fig. S1.** The receiver-operating characteristic (ROC) curve for C-reactive protein (CRP in mg/dL, 1A and CRP velocity (CRP /time in hours from symptoms onset, 1B) to diagnose bacterial infections in the neonatal intensive care unit.

**Additional file 2:** **Fig. S2.** CRP velocity (CRP/time in hours from symptoms, 2A) and CRP decrease rate (CRP velocity at time 1/CRP velocity at time 2, 2B), for bacterial and non-bacterial groups.

## Data Availability

The datasets used and/or analyzed during the current study are available from the corresponding author on reasonable request.

## Abbreviations

CRP: C-reactive protein
NICU: Neonatal intensive care unit
ROC curve: Receiver-operating characteristic
LRs: Likelihood ratios
CONS: Coagulase-negative s*taphylococci*
ED: emergency department
MRSA: Methicillin-resistant *S.aureus*
GI: Gastrointestinal
AUC: Area under the ROC curve
CDC: Centers for Disease Control and Prevention
NHSN: National Healthcare Safety Network
