# Supplementary figures and images for: Time from symptom onset may influence C-reactive protein utility in the diagnosis of bacterial infections in the NICU
Source: BMC Pediatr. 2022 Dec 14;22:715. doi: 10.1186/s12887-022-03783-4 (PMC9749206; doi:10.1186/s12887-022-03783-4)

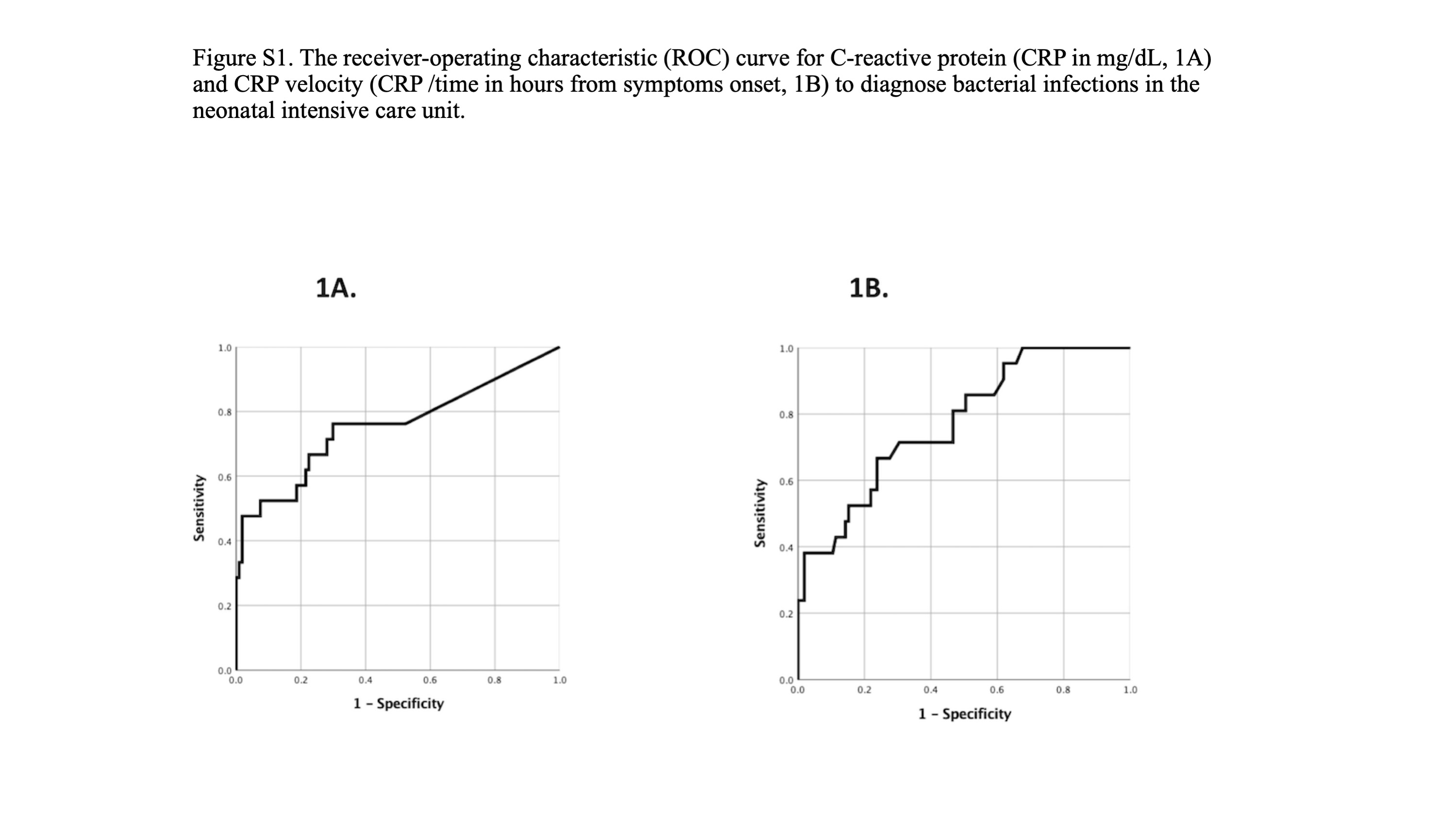

Supplement: Supplementary file 1 — Additional file 1: Fig. S1. The receiver-operating characteristic (ROC) curve for C-reactive protein (CRP in mg/dL, 1A and CRP velocity (CRP /time in hours from symptoms onset, 1B) to diagnose bacterial infections in the neonatal intensive care unit. [file 12887_2022_3783_MOESM1_ESM.jpg]

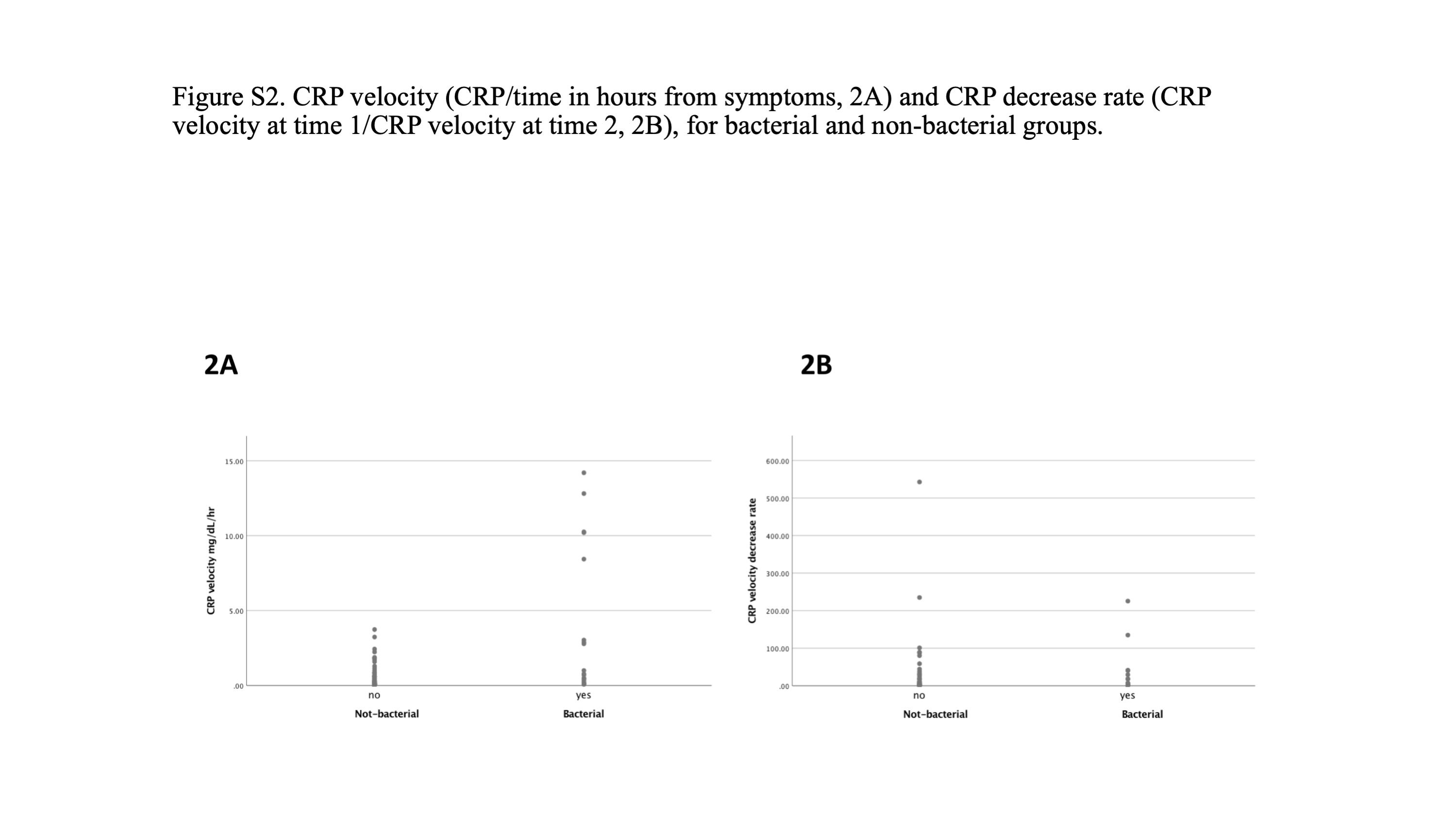

Supplement: Supplementary file 2 — Additional file 2: Fig. S2. CRP velocity (CRP/time in hours from symptoms, 2A) and CRP decrease rate (CRP velocity at time 1/CRP velocity at time 2, 2B), for bacterial and non-bacterial groups. [file 12887_2022_3783_MOESM2_ESM.jpg]
